# Brain microhemorrhages in adult-onset Still’s disease: A case report

**DOI:** 10.1097/MD.0000000000042127

**Published:** 2025-04-18

**Authors:** Jung Youn Kim, Hye Jeong Choi, Sang Heum Kim

**Affiliations:** a Department of Radiology, CHA Bundang Medical Center, CHA University, Seongnam, Republic of Korea.

**Keywords:** adult-onset Still’s disease (AOSD), microhemorrhages, seizure, SWI (susceptibility-weighted imaging)

## Abstract

**Rationale:**

Adult-onset Still’s disease (AOSD) is a rare, systemic inflammatory disease. Neurological involvement in AOSD has rarely been reported. In this case report, we describe a 44-year-old male patient with AOSD who presented with seizures and scattered microhemorrhages on brain magnetic resonance imaging (MRI). We recommend brain MRI evaluation, including susceptibility-weighted imaging (SWI), for patients with AOSD who exhibit neurological symptoms.

**Patient concerns:**

A 44-year-old male patient presented to the clinic for a strikingly high fever, sore throat and myalgia that persisted for 3 weeks. The vital signs were stabilized upon empirical treatment after admission. On the 11th day after admission, he showed high spiking fever (up to 39.9°C) and generalized tonic-colonic seizure for 5 minutes. Extremely elevated serum ferritin level (≥16,500 ng/mL) was noted over the detection limit. Brain MRI with SWI showed multiple tiny dark signals in the splenium of the corpus and the subcortical white matter.

**Diagnoses:**

AOSD was diagnosed based on the Yamaguchi criteria.

**Interventions:**

Nonsteroidal anti-inflammatory drugs (NSAID), Tacrolimus, Hydroxychloroquine, and anticonvulsants.

**Outcomes:**

The patient recovered and the seizures did not recur.

**Lessons:**

Brain MRI evaluation, including SWI, is essential for patients with AOSD who present with neurological symptoms.

## 1. Introduction

Adult-onset Still’s disease (AOSD) is a rare systemic inflammatory disorder characterized by a triad of spiking high fever, evanescent rash and arthralgia.^[1]^ The symptoms may include sore throat, myalgia, lymphadenopathy, and hepatosplenomegaly.^[2]^ Neurological involvement in AOSD is uncommon, with seizures being an extremely rare manifestation.^[1,3–5]^ There have been few reports in the literature on brain MRI findings showing multiple microhemorrhages in the subcortical white matter (WM) on susceptibility-weighted imaging (SWI) in AOSD patients.^[6]^ Herein, we present the case of a male patient with AOSD who exhibited seizures and corresponding brain MRI findings.

## 2. Case presentation

**Figure 1.**
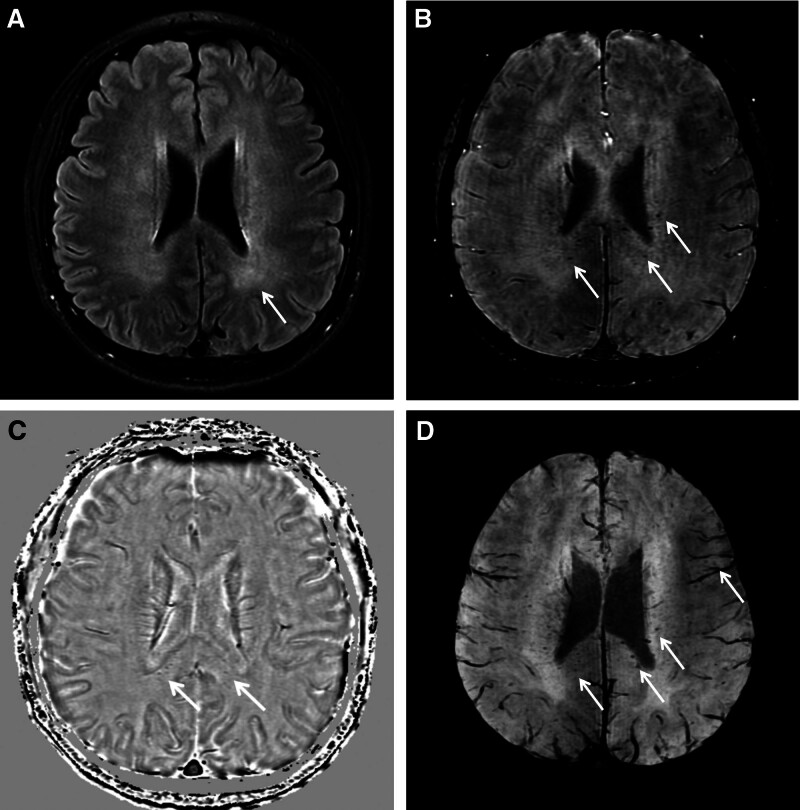
MRI of adult-onset Still’s disease patient seizure. (A) Axial spin-echo T2-FLAIR imaging with fat suppression shows diffuse periventricular white matter (WM) signal changes. (B) On SWI, multiple microhemorrhages (arrows) are scattered in the periventricular WM, especially in the splenium of the corpus callosum. (C) High-pass filtered image reveals tiny hemorrhagic signals in the WM identifying the component of the signals. (D) The minimum intensity projection of 5 SWI images displays hemorrhagic signals frequently in the periventricular WM (especially in the splenium of the corpus callosum) and even in the subcortical WM of the left frontal lobe (arrows). FLAIR = fluid-attenuated inversion recovery, SWI = susceptibility-weighted imaging, WM = white matter.

A previously healthy 44-year-old male presented to the clinic with strikingly high fever, sore throat, and myalgia that had persisted for 3 weeks. The patient had a non-pruritic skin rash on both hands and arthralgia in the left foot. There had no history of trauma, travel, sick contact, family, or medical history. The patient denied any oral ulcers, morning stiffness, or ocular symptoms. The vital signs were as follows: body temperature, 38°C, blood pressure, 120/80 mm Hg; and heart rate, 70 beats/min. Laboratory tests revealed peripheral leukocytosis, with a white blood cell count of 18,100/mm^3^ (86% neutrophils) and elevated levels of inflammatory markers, including erythrocyte sedimentation rate (ESR; 81.3 mm/h) and C-reactive protein (CRP; 21.36 mg/dL) levels. Abnormal liver function with elevated alanine aminotransferase (ALT) and aspartate aminotransferase (AST) levels of 75 IU/L and 144 IU/L, respectively, was noted. Coagulation tests revealed mild prolongation of prothrombin time (PT) to 15.1 second (reference range 10.0–12.7) and normal activated partial thromboplastin time (aPTT). The patient’s renal profile was normal. Antinuclear antibodies (ANA) were positive, with a middle titer of 1/320. The anti-Ro (SSA) and anti-La (SSB) antibodies, anticardiolipin antibodies (ACA), anti cytoplasmic antibodies (ANCA), anti-cyclic citrullinated peptide antibodies (ACCP), and rheumatoid factor (RF) tests were all negative. Blood and urine tests and cultures revealed no evidence of infection. The serum ferritin level markedly increased to 11,202 ng/mL (reference interval 22–322). Abdominal computed tomography revealed splenomegaly. AOSD was diagnosed based on Yamaguchi criteria.^[2]^ Steroids and non anti-inflammatory drugs (NSAID) were initiated. The patient’s vital signs stabilized upon empirical treatment after admission. On the 11th day after admission, he showed high spiking fever (up to 39.9°C) and generalized tonic-colonic seizure for 5 minutes. Extremely elevated serum ferritin levels (≥16,500 ng/mL) were noted over the detection limit. Neurological and systemic examinations revealed a mental state confused with sedation. Routine electroencephalogram showed no evidence of seizure activity. 3T magnetic resonance imaging (MRI) scan was performed. In T2-FLAIR (fluid-attenuated inversion recovery) imaging with fat suppression revealed diffuse WM signal alterations mainly in the periventricular WM (Fig. 1A). On SWI, the high-pass filtered image and minimal intensity projection image showed multiple tiny dark signals in the splenium of the corpus and subcortical WM (Fig 1B–D). Tacrolimus and hydroxychloroquine were also administered. He was recommended 2000 mg levetiracetam as a loading dose, followed by 750 mg twice daily; there were no more seizures during the follow-up, and he recovered to an alert mental state after 5 days of follow-up.

## 3. Discussion

AOSD is a rare systemic inflammatory disease.^[1]^ Diagnosis is often made by excluding other infectious, neoplastic, and autoimmune diseases, and Yamaguchi’s criteria are well known.^[1,2]^ AOSD is characterized by high fever, arthralgia, rash, sore throat, lymphadenopathy, hepatomegaly, and splenomegaly.^[2]^ Laboratory tests show neutrophilic leukocytosis, an increase in ESR/CRP, anemia, or thrombocytosis that reflects nonspecific systemic inflammation, and there is no single discriminative diagnostic test.^[1]^ High serum ferritin levels are notably higher in patients with AOSD than in those with other inflammatory, infectious, autoimmune, or neoplastic diseases. Some authors suggest that a threshold of 1000 ng/mL, which is 5 times the normal value, is indicative of AOSD.^[1]^ In addition, serum ferritin level is considered a valuable marker of disease activity in AOSD.^[1,7]^

Neurological involvement in patients with AOSD is less frequently reported in the literature.^[1,3–5]^ Reported to include intracranial arteriopathy, ischemic stroke, reversible posterior leukoencephalopathy syndrome, aseptic meningitis, encephalitis, cranial nerve palsy, demyelinating polyneuropathy, and Guillain-Barre syndrome.^[1,3–5,7]^ Fatal cerebral edema has been reported in 2 young patients who underwent brain imaging and autopsy.^[6,8]^ In 1 study, multiple fibrin thrombi disseminated in small vessels in the brain with significant edema were observed in autopsy specimens.^[8]^ Another case showed isolated clusters of activated macrophages and microglia throughout the brain during autopsy.^[6]^ The computed tomography and MRI imaging revealed diffuse cerebral edema and hypoxic–ischemic injury.^[6]^ However, they did not obtain hemorrhage-specific sequences, such as SWI or T2* gradient recalled echo.^[6]^ In our case, SWI showed multiple tiny dark signals in the corpus callosum and subcortical WM, suggesting brain microhemorrhage. The pathogenesis of these microhemorrhages in patients with AOSD can be explained by the involvement of thrombotic microangiopathy and microthrombus formation.^[9]^ SWI provides highly sensitive information about microhemorrhages, which are subtle changes in brain tissue.^[10]^

The therapeutic strategy for AOSD is based on empirical treatment.^[11]^ NSAID and corticosteroids are the first-line treatments for this condition.^[11]^In patients dependent on steroids, a disease-modifying antirheumatic drug should be introduced to facilitate the gradual reduction of glucocorticoids and minimize the risk of long-term toxicity. Biological therapies such as interleukin (IL)-1 and IL-6 inhibitors are effective in severe or steroid-dependent cases.^[11,12]^

## 4. Conclusion

This case indicates that neurological symptoms may occur in patients with AOSD. Spiking fever and high ferritin levels suggest disease aggravation during the course of AOSD. For patients with AOSD exhibiting neurological symptoms, brain MRI is recommended, including SWI, which is highly sensitive for the detection of microhemorrhages, and conventional imaging to elucidate brain involvement in AOSD and to exclude other diseases.

## Author contributions

**Conceptualization:** Jung Youn Kim, Hye Jeong Choi.

**Data curation:** Jung Youn Kim, Hye Jeong Choi, Sang Heum Kim.

**Investigation:** Jung Youn Kim, Hye Jeong Choi, Sang Heum Kim.

**Supervision:** Hye Jeong Choi.

**Writing – original draft:** Jung Youn Kim.

**Writing – review & editing:** Hye Jeong Choi.
